# Osteoblastic differentiation and stress response of human mesenchymal stem cells exposed to alternating current electric fields

**DOI:** 10.1186/1475-925X-10-9

**Published:** 2011-01-26

**Authors:** Marie Hronik-Tupaj, William L Rice, Mark Cronin-Golomb, David L Kaplan, Irene Georgakoudi

**Affiliations:** 1 Department of Biomedical Engineering,4 Colby Street, Science & Technology Center, Tufts University, Medford, MA 02155 USA

## Abstract

**Background:**

Electric fields are integral to many biological events, from maintaining cellular homeostasis to embryonic development to healing. The application of electric fields offers substantial therapeutic potential, while optimal dosing regimens and the underlying mechanisms responsible for the positive clinical impact are poorly understood.

**Methods:**

The purpose of this study was to track the differentiation profile and stress response of human bone marrow derived mesenchymal stem cells (hMSCs) undergoing osteogenic differentiation during exposure to a 20 mV/cm, 60 kHz electric field. Morphological and biochemical changes were imaged using endogenous two-photon excited fluorescence (TPEF) and quantitatively assessed through eccentricity calculations and extraction of the redox ratio from NADH, FAD and lipofuscin contributions. Real time reverse transcriptase-polymerase chain reactions (RT-PCR) were used to track osteogenic differentiation markers, namely alkaline phosphatase (ALP) and collagen type 1 (col1), and stress response markers, such as heat shock protein 27 (hsp27) and heat shock protein 70 (hsp70). Comparisons of collagen deposition between the stimulated hMSCs and controls were examined through second harmonic generation (SHG) imaging.

**Results:**

Quantitative differences in cell morphology, as described through an eccentricity ratio, were found on days 2 and days 5 (p < 0.05) in samples exposed to the electric field. A delayed but two fold increase in ALP and col1 transcript was detected by week 2 (p < 0.05) in differentiating hMSCs exposed to an electric field in comparison to the nonstimulated controls. Upregulation in stress marker, hsp27, and type 1 collagen deposition were correlated with this response. Increases in NADH, FAD, and lipofuscin were traced in the stimulation group during the first week of field exposure with differences statistically significant on day 10 (p < 0.05). Changes in hsp27 expression correlate well with changes in lipofuscin detected in the stimulation group, suggesting a connection with oxidative stress. Both differentiation factors and electrical stimulation improved hMSC differentiation potential to bone based on calcium deposition on day 28.

**Conclusions:**

Electrical stimulation is a useful tool to improve hMSC osteogenic differentiation, while heat shock proteins may reveal underlying mechanisms, and optical non-invasive imaging may be used to monitor the induced morphological and biochemical changes.

## Background

In the United States, fractures account for 5.6 million annual musculoskeletal conditions [1]. After one year post-injury, 5-10% of bone fractures show impaired healing and require additional orthopedic intervention [1]. The clinical practice of orthopedics has empirically used many techniques to establish an optimal environment for skeletal repair [2,3], including grafting, casting, and splinting methods [2]. For more severe fractures, treatments include load-bearing techniques that range from mechanical stress to ultrasound [4,5]. Musculoskeletal tissues, including bone and cartilage, respond to biophysical inputs such as electric and electromagnetic fields [6]. Biophysical stimulation is advantageous over pharmacological or chemical therapeutics due to the absence of local toxicity in the surrounding tissue or adverse systemic reactions. To apply biophysical stimulation for clinical use, non-invasive and implantable electromagnetic devices are used, such as bone growth stimulators [7,8]. Non-invasive devices may allow for patient comfort, while implantable devices ensure patient compliance [9,10]. Clinical devices employ pulsed electromagnetic fields (PEMF), or direct currents (DC) for healing nonunions and spinal fusions[11,12]. For example, in one clinical study, 76% of recalcitrant non-unions were healed during treatment employing capacitively coupled (CC) alternating current electric fields[13].

As electromagnetic stimulators are clinically successful for healing bone fractures, we aimed to further examine the impact and mechanisms of electric fields on bone related outcomes through utilizing a human stem cell source. hMSCs have proved to be a competent cell source for tissue engineering applications including bone, cartilage, and adipose tissue regeneration. Furthermore, mesenchymal stem cells have demonstrated a positive response to physical forces [14]. For example, ossicles that are stimulated solely in the mesenchymal stage yield calcium content commensurate with ossicles that are stimulated throughout development [15]. Thus these cells may be more sensitive to electromagnetic field stimulation during bone formation [15].

In the present study, we hypothesized that a 20 mV/cm 60 kHz electric field applied for 40 minutes daily would increase biomarker expression and stress response on hMSCs, and would decrease the time to osteogenic differentiation. The rationale for choosing AC electric current at 60 kHz, 20 mV was based on a series of previous studies that reported increases in osteogenic and chondrogentic differentiation markers, such as TGF-β_1_, type 2 collagen, proteoglycan, bone morphogenetic proteins, as well as cell proliferation at this strength [16-20]. A 60 kHz, 20 mV/cm electric field strength was used for all studies. Initial electric field effects on differentiating mesenchymal stem cells were observed by examining cell morphological and biochemical changes using non-destructive two-photon excited fluorescence (TPEF) imaging. Metabolic profiles of the cells were tracked to provide insight into whether an increased charge in the surrounding environment alters metabolic pathways, which could in turn impact hMSC osteogenic differentiation and bone healing. Our electric field setup was modeled as two electrodes in parallel separated by a 1 cm distance. Electrodes were in direct ohmic contact with cell culture media. During stimulation, ionic current was transferred through the culture media across the chamber. To provide insight into the rate and extent of osteogenic differentiation in a low frequency AC electric field, early and mid stage differentiation markers, alkaline phosphatase (ALP) and collagen type I (col1) were assessed at the transcript level. We examined stress responses with heat shock protein 27 (hsp27) and heat shock protein 70 (hsp70), due to the ability of these markers to impact osteogenic differentiation and cell metabolism [21-23]. Indeed, heat shock protein responses increased in response to stimulation and were correlated with bone differentiation markers. Finally, for the purpose of examining post-translational col type 1 expression and complete bone differentiation, type 1 collagen and calcium deposition were assessed through second harmonic generation imaging and Alizarin Red staining, respectively.

## Methods

### Human mesenchymal stem cell culture

hMSCs (Tulane University, New Orleans, LA) were from male donors < 25 years of age and plated for expansion at 5,000 cells/cm^2 ^in T175 flasks with Dulbecco's Modified Eagle Medium (DMEM) containing 1 g/L D-glucose, and 110 mg/L sodium pyruvate (Invitrogen Corp, Grand Island, NY). DMEM was supplemented with 584 mg/L L-glutamine, 3,500 mg/L D-glucose, 10% fetal bovine serum, 1% penicillin-streptomycin, 0.25 mg/mL fungizone, 0.1 mM nonessential amino acids, and 1 ng/mL basic fibroblast growth factor (bFGF) containing 10 mM Tris at pH 7.6 and 0.1% bovine serum albumin (BSA) (Invitrogen Corp, Grand Island, NY). Cell culture medium was changed two times per week. All experiments used hMSC passages between P2-P6.

### Osteogenic differentiation

Osteogenic differentiation medium included standard hMSC culture medium as listed above with the exception of 1 ng/mL bFGF. In addition, 0.05 mM L-ascorbic acid 2-phosphate (Sigma-Aldrich, St. Louis, MO), 1,000 nM dexamethasone-water soluble (Sigma-Aldrich, St. Louis, MO), 10 mM glycerol 2-phosphate disodium salt hydrate (Sigma-Aldrich, St. Louis, MO), and 100 ng/mL bone morphogenetic protein (BMP)-2 (gift from Wyeth, Madison, NJ) was added. Differentiation medium was changed two times per week.

### Electrical stimulation chamber design

**Figure 1:**
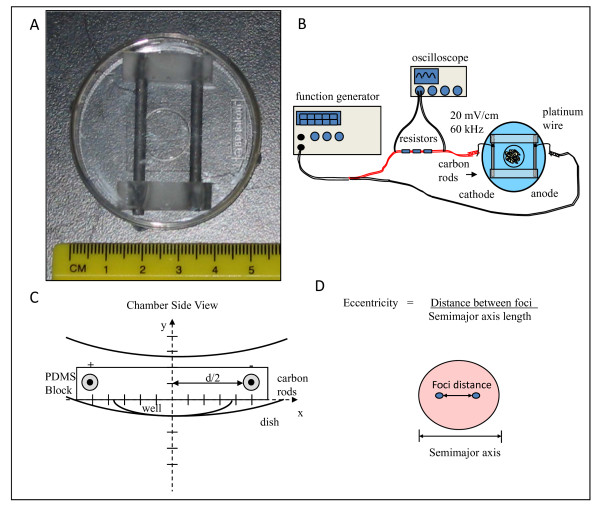
**Chamber Design, Electric Field Setup and Strength Calculations**. (a) Carbon electrode chamber design for capacitvely coupled field (b) Electric field setup including function generator for power supply and oscilloscope for electric field strength verification (c) Chamber side view outlining chamber dimensions for field strength calculations (d) Cell dimensions for morphological assessment.

The electrical stimulation chamber was modified from previous designs to interface with imaging equipment [24]. Briefly, two 5 mm long carbon rods (Ladd Research, Williston, VT), 3 mm in diameter were separated using two 25×7×7 mm polydimethylsiloxane (PDMS) blocks. PDMS blocks were custom made using a Sylgard 184 Silicone Elastomer Kit (Ellsworth Adhesives, Germantown, WI) in a 10:1 (w/w) ratio of base to curing agent. Two 2 mm diameter holes having a distance of 10 mm apart were punched into the PDMS block. A carbon rod was pushed through each hole. A second PDMS block was placed on the opposite ends of the carbon rods for support. Two 8 cm long platinum wires with 99.995% purity (Surepure Chemetals, Florham Park, NJ) were secured between each carbon rod and PDMS support to create a strong connection with the rod. Following assembly, PDMS supports, carbon rods, and platinum wire were autoclaved then placed in a 50 mm diameter poly-d-lysine coated glass bottom Falcon Dish that contained a 14 mm diameter glass coverslip with a thickness of 0.16-0.18 mm (MatTek Corporation, Ashland, MA) (Figure 1a). For a voltage source, a TENMA Universal Test Center 72 -1005 Function Generator (TENMA Test Equipment, Springboro, OH) was connected to the stimulation chamber's platinum wires via alligator clips and 18 gauge copper wires (resistivity ρ = 1.724 × 10^-8 ^ohm m). Three 220 Ω resistors were placed on one of the copper wire leads in series (Figure 1b).

### Electric field strength calculations

Assuming both carbon rods in the chamber are of equal diameter and have a uniform surface charge density, derivations were made using a form of Gauss's Law that calculates electric field strength at any distance, r, from an infinite line charge (equation 1).

(1) $$E=\frac{1}{2\pi \varepsilon }\left(\frac{\rho }{r}\right)$$

To adapt Gauss's Law for our own chamber model, we first converted equation 1 from cylindrical coordinates to Cartesian Coordinates and offset each rod by a distance of ±$\frac{d}{2}$. Electric field intensity at any point (x,y) in the stimulation chamber is the summation of individual charge contributions from both the positively and negatively charged rods (equation 2).

(2) $$E_{x}=\frac{V_{ab}}{2\ln\left(\frac{a}{d-a}\right)}\left[\frac{x-\frac{d}{2}}{{\left(x-\frac{d}{2}\right)}^{2}+y^{2}}-\frac{x+\frac{d}{2}}{{\left(x+\frac{d}{2}\right)}^{2}+y^{2}}\right]$$

V_ab _is the voltage potential measured across the carbon rods, a is the carbon rod radius, d is the distance between the carbon rods, x is the distance in the x direction of the cells from the center of the carbon rods, and y is the distance of cells from the charged rods in the y direction (Figure 1c). For a complete derivation see Appendix. To ensure that 20 mV/cm electric field strength (E_x_) reached the cells at any point in the glass well, equation 2 was used to identify the voltage potential (V_ab_) needed at the carbon rods. A 60MHz 2213A Tektonix oscilloscope (Beaverton, OH) was used to measure the voltage across the carbon electrodes in culture media and ensure that a 20 mV/cm potential strength reached the cells inside the chamber.

### Experimental design

The experimental setup included two study groups; a control group consisting of hMSCs in osteogenic differentiation medium and a stimulation group consisting of hMSCs with differentiation medium and the applied electrical stimulation. All differences in osteogenic differentiating hMSCs exposed to the applied electric field were compared to the control group consisting of osteogenic differentiating hMSCs only. Early gene expression markers and calcium content of a positive hMSC osteoblast control group and negative hMSC adipogenic control groups have been reported previously in our lab [25]. Stem cells were initially seeded at a density 50,000 cells/cm^2 ^in 150 uL of culture media on individual glass bottom dishes or individual glass bottom dishes with stimulation chambers. Cells were allowed 45 minutes to attach to the bottom of the dishes and then 2-4 mL of culture or differentiation medium were added per sample to the glass well. All samples were kept at 37°C and 5% CO_2_. Each day, hMSC samples in the stimulation chambers were exposed to field strengths 20 mV/cm oscillating at 60 kHz. The 60 kHz frequency was chosen as previous studies utilizing alternating current fields reported increases in osteogenic and chondrogenic differentiation markers, such as TGF-β_1_, type 2 collagen, proteoglycan, as well as cell proliferation [17-19]. The applied signal was a symmetrical sine wave applied 40 minutes daily for 28 days. Prior to daily stimulation the voltage potential and frequency were measured over the 220 Ω resistors using an oscilloscope to ensure that the applied field strength was the same each day and the same over each sample. A 100 μA current was calculated through the 220 Ω resistors according to Ohm's Law. Autoclave paper was placed under each chamber before stimulation to prevent any additional unwanted electrical connections. All electric field stimulations were applied in a laminar flow hood. For consistency, the control samples were placed at room temperature during the time of stimulation. On days 5, 10, 15, and 20 stimulation and control dishes were sacrificed for real time- polymerase chain reaction (RT-PCR). Cellular activity measurements were taken using AlamarBlue on days 5, 10, 15, 20. Two-photon excited fluorescence (TPEF) and second harmonic generation (SHG) images were taken at day 2 and on every fifth day to assess morphological changes, collagen deposition, and metabolic activity. On day 28, Alizarin Red stain was applied to assess calcium content In previous studies, mineralization has been reported within 3-4 weeks of hMSCs undergoing osteogenic differentiation with the addition of (BMP)-2 to the differentiation media [26,27]. The experiment ran for 28 days as this was sufficient time to observe hMSC osteogenic differentiation with the addition of osteogenic differentiation medium and (BMP)-2.

### Morphology

Two-photon excited fluorescence (TPEF) images based on endogenous fluorescence emission were acquired using a two-photon ready Leica DM IRE2 confocal microscope (Leica, Wetzlar, Germany) and equipped with a Mai Tai solid state tunable (710-920 nm) Ti:sapphire laser (Spectra Physics, Mountain View, CA), emitting 100 fs pulses at a rate of 80 MHz. TPEF images were taken at 755, 800, and 860 nm excitation and 455 nm ± 35 nm and 525 nm ± 25 nm emission using two non-descanned detectors. Images were acquired with a water immersion, 63×, numerical aperture of 1.2 objective. Incident laser power was approximately 12.5 mW at 755 nm and 5.2 mW at 860 nm. Three areas per sample were imaged from two samples per group. Cell shape and morphological changes were quantitatively described through the eccentricity ratio, defined as the distance between the foci of an ellipse over the semi-major axis length (Figure 1d). As the eccentricity ratio approaches zero, the cell shape becomes rounder; while the ratio is closer to 1 as the cells become more elongated. Eccentricity calculations were done using a program that was created in MATLAB (MathWorks, Natick, MA). Morphological changes were graphed as average eccentricity value vs. time. Thirty cells were assessed at each time point from each of the groups (i.e. control and stimulated). Data are reported as the mean ± one standard deviation of the mean due to the large sample size (n = 30).

### Real time reverse transcriptase - polymerase chain reaction

mRNA was extracted using trizol and collected using the Qiagen RNEasy Extraction kit (Qiagen, Valencia, CA). Samples were stored at -80°C until assayed. cDNA was amplified with the TaqMan Universal PCR Master Mix (Applied Biosystems, Foster City, CA) and the ABI Prism 7000 Sequence Detection System (Applied Biosystems). RT-PCR determined mRNA expression of osteogenic differentiation markers, alkaline phosphatase (ALP Assay ID #: Hs00240993_m1), and collagen type 1 (Col1 Assay ID #: Hs00164004_a1), and stress response markers, heat shock protein 27 (hsp27 Assay ID #: Hs00356629_g1) and heat shock protein 70 (hsp70 Assay ID #: Hs00271244_s1). Assays on demand were purchased through Applied Biosystems (Carlsbad, CA). Relative gene expression was normalized to the housekeeping gene GAPDH (Assay ID #: Hs99999905_m1) and calculated using the formula 2^(Ct value of GAPDH - Ct value of gene of interest) ^as previously used in our lab [28] and recommended by the manufacturer (Perkin Elmer User Bulletin #2, Applied Biosystems, Foster City, CA). The threshold cycle (Ct) was selected in the linear range of fluorescence for all genes.

### Metabolic activity

#### AlamarBlue

For the purpose of assessing changes and differences in cellular activity, the control and stimulation groups were assayed every 5 days using AlamarBlue (Invitrogen Corp, Grand Island, NY). Briefly, a 9:1 (v/v) dilution of AlamarBlue to cell culture medium was added directly to the samples, then incubated for 2.5 hours. Following incubation, three 100 uL replicates of media containing AlamarBlue was pipetted from each sample into a black 96 well plate. Fluorescence readings were taken using a plate reader at 560 nm excitation, 590 nm emission. Arbitrary units are determined as relative units of fluorescence intensity from the reduction of resazurin found in AlamarBlue to red fluorescent resorufin in the presence of metabolically active cells. Since AlamarBlue is nontoxic to cells, media that contained AlamarBlue was replaced with fresh differentiation medium following each reading. The same samples were measured at each time point. Excel was used to plot metabolic activity as a function of arbitrary units (AU) vs. time. Graphs were normalized to the fluorescence reading of the Alamar solution alone. Sample size per group per time point was n = 3.

#### Biochemical characterization

Quantities of NADH, FAD, and lipofuscin found in osteogenically differentiating hMSCs were determined from analysis of endogenous TPEF images acquired on day 0 and every five days using a quantitative approach described in detail recently [29]. Briefly, images were taken at 860 and 755 nm excitation with emission filters centered at 525 and 455 nm as described in the morphology section. Images were filtered to remove noise and saturated pixels. The contribution from lipofuscin was determined as the fluorescence signal from the 455 nm channel at 860 nm excitation. For identifying the FAD contribution, a lipofuscin mask was created and multiplied with the fluorescence signal at 860 nm excitation, 525 emission; signal residing in pixels outside of the mask were assigned to FAD. NADH was calculated similarly by multiplying the lipofuscin mask with the 755 nm excitation, 455 emission channel, and considering the signal not emanating from pixels within the mask. The mask is the location of lipofuscin fluorescence as determined from data in the 455 nm channel at 860 nm excitation [29]. The mask was calculated as all pixels above a 10% threshold in the image [29]. This approach is reasonable because lipofuscin accumulates primarily in lysosomes, while NADH and FAD fluorescence originates predominantly from mitochondria [29]. Metabolic activity was determined through calculating the redox ratio as Redox Ratio = FAD/ NADH. Decreases in the redox ratio yield increased metabolic activity, while a higher redox ratio reveals decreased metabolic activity. Unpaired t-tests were performed between the treated and untreated groups at each time point and between each time point per group. Sample size per group per time point was n = 3.

### Protein deposition

Images for observing type 1 collagen deposition were acquired through a non-destructive, non-linear optical scattering process, second harmonic generation (SHG) [30]. SHG images were acquired at 800 nm excitation in the forward scattering direction with the same system as the TPEF images using a 400 nm ± 10 nm emission filter (Chroma, Rockingham, VT). Following imaging, collagen deposition was quantified through a fully automated adaptive threshold-based program written in MATLAB (MathWorks, Natick, MA), described in detail previously [31]. Sample size was n = 3. The reported collagen fiber density refers to the percentage of pixels that are positive for SHG signal relative to the total number of pixels imaged within a field (i.e. a value of 0.1 corresponds to 10% of the pixels having SHG signal).

### Calcium staining

To determine the presence of calcium, samples were washed with phosphate buffered saline, then fixed for 15 minutes using 1% formaldehyde. Following fixation, samples from the control and stimulation groups were rinsed twice with water and soaked in an Alizarin Red solution for 10 minutes. Alizarin Red S (Sigma-Aldrich, St. Louis, MO) was weighed and diluted in deionized water to a final concentration of 0.8 g:40 mL (w/v). The solution pH was adjusted to 4.2. Samples were imaged in water using a Zeiss Axiovert S100 phase contrast microscope.

### Statistics

Data are reported as the mean ± one standard deviation unless otherwise noted. Data were analyzed using an unpaired two-tailed student t-test, assuming equal variance with a 95% confidence interval. A p-value of < 0.05 was considered statistically significant unless otherwise noted. In addition, for reporting a large number of comparisons between time points, the Bonferroni Correction factor was applied to correct for multiple comparisons of artifacts. For determining statistical significance using the Bonferroni Correction factor, alpha was set as α = 0.05/ 10 = 0.005.

## Results

### Electric field effects on cell morphology

**Figure 2:**
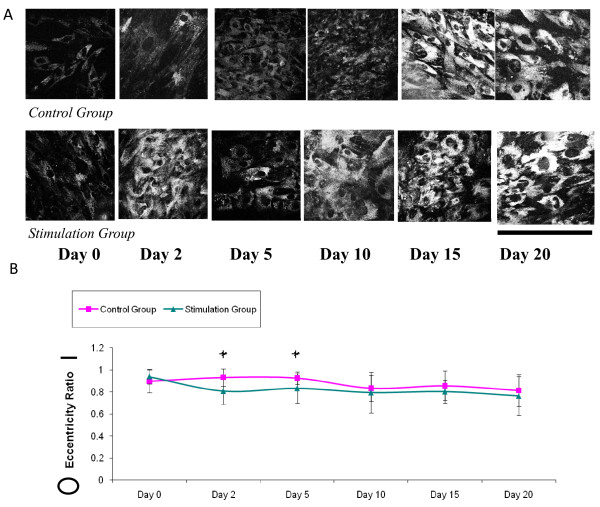
**Morphological Changes during Osteogenic Stem Cell Differentiation**. (a) TPEF images showing morphological changes on days 0, 2, 5, 10, 15, 20 of hMSCs undergoing ostegenic differentiation. Images are of groups exposed and unexposed to an electric field (b) Morphological changes quantified by an eccentricity ratio and graphed vs. time. Statistical differences in cell shape between the control and stimulation groups are noted by an asterisk at day 2 and 5 (p < 0.05). The means of the two groups are statistically different despite the overlap of the error bars, because the sample size of cells circled is large (n = 30).

On day 0 following initial stem cell seeding and attachment, TPEF images were taken for baseline morphological comparisons (Figure 2a). Cells in the control and stimulation groups appeared thin and elongated, a characteristic of undifferentiated hMSCs. Throughout the experiment, both groups exhibited a distinct change in cellular phenotype. At the first two time points, stem cells exposed to an electric field appeared rounder than those in differentiation medium only and had an eccentricity value (Figure 2b) that was statistically lower than the control group (p < 0.05). By day 10, the cells in both groups exhibited a circular morphology. Quantitative morphological measures of eccentricity over 20 days are graphed in Figure 2b.

### Osteogenic markers

**Figure 3:**
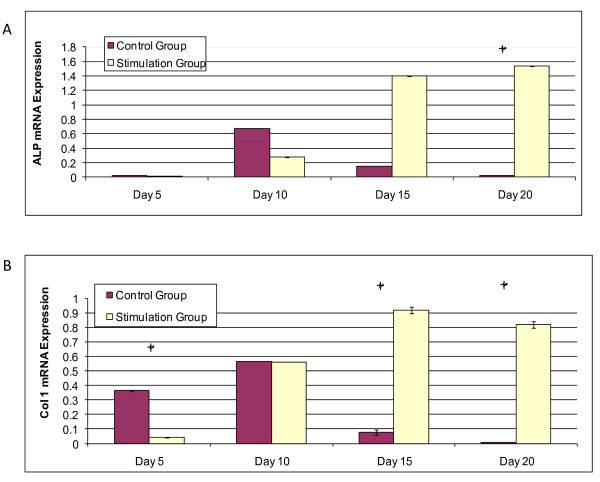
**mRNA Expression Levels during Osteogenic Differentiation**. (a) ALP mRNA expression relative to GAPDH vs. time. (b) Col1 mRNA expression relative to GAPDH vs. time. Statistical significance between groups is marked by an asterisk (p < 0.05).

Osteogenically differentiating hMSCs revealed overexpression of the early bone marker alkaline phosphatase (ALP) and mid marker type 1 collagen (col1). In the short term, on days 5 and 10, ALP and col 1 mRNA expression increased in the control group (Figure 3a, 3b) (p < 0.05); osteogenically differentiating stem cells in the stimulation group showed enhanced ALP and col1 at later time points, days 15 and 20 (Figure 3a, 3b) with statistically significant differences in ALP expression at day 20 and col1 at day 15 and day 20. During the second week, ALP and col1 upregulation of the osteogenic differentiating stem cells in the stimulation group were two times greater than expression in the control group at day 10. At the end of week 2, the stimulation group had ALP and col1 upregulation statistically higher than the control (p < 0.05).

### Stress response markers

**Figure 4:**
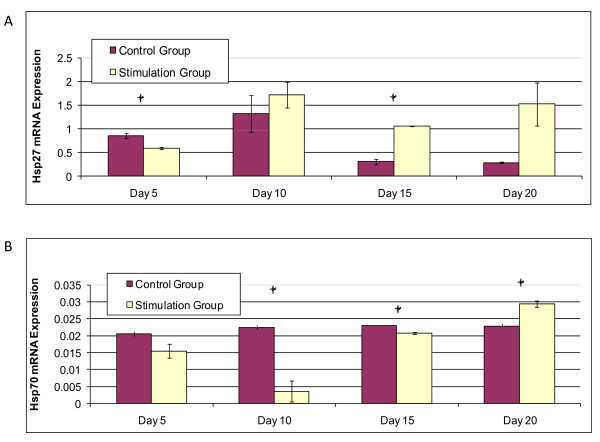
**Heat Shock mRNA Expression**. (a) Hsp27 mRNA expression relative to GAPDH vs. time. (b) Hsp70 mRNA expression relative to GAPDH vs. time. Statistical significance between groups is marked by an asterisk (p < 0.05).

Heat shock protein 27 (hsp27), a stress response marker involved in osteogenic differentiation, was upregulated in the stimulation group on days 10, 15, and 20 when compared to the control group (Figure 4a). Hsp27 upregulation was statistically significant compared to the control group on day 15 (p < 0.05). Hsp70, a protein involved in cellular metabolism, was upregulated in the stimulation group at day 20 (p < 0.05), however consistent upregulation compared with controls was not observed (Figure 4b). In fact, Hsp70 expression was higher for the control groups on day 10 and 15.

### Cellular and Metabolic activity changes

**Figure 5:**
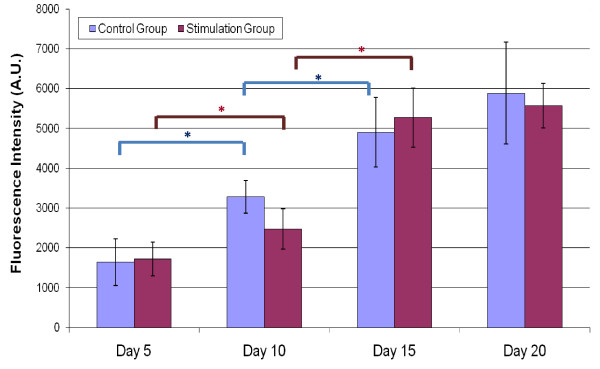
**Cellular Activity**. Normalized metabolic activity graphed as fluorescence intensity in arbitrary units (A.U.) vs Time. Statistical differences between time points are marked by a blue asterisk (p < 0.05) for the control group or a red asterisk (p < 0.05) for the stimulation group. Statistical differences are seen in the control group between day 5 - day 10 and between day 10 - day 15. Statistical differences are also seen in the stimulation group between day 5 - day 10 and between day 10 - day 15.

Increases in cellular activity were found using Alamar blue staining in both groups over the 20 day differentiation period (Figure 5). Increased cellular activity (p < 0.05) was identified in the control and stimulation groups from day 5 to day 10 and from day 10 to day 15. There were not any significant changes in cellular activity identified between the two groups either on day 15 or day 20.

**Figure 6:**
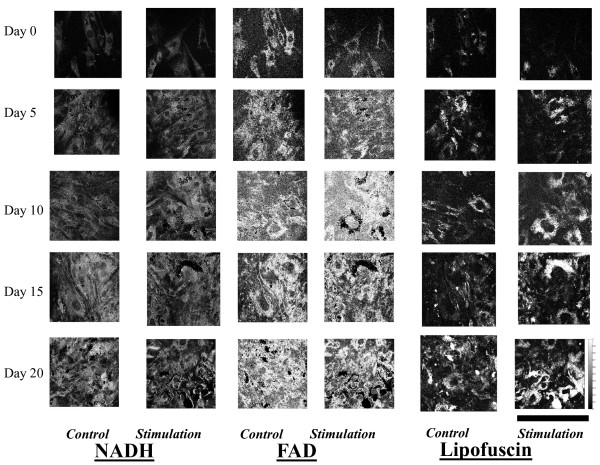
**NADH, FAD, Lipofuscin**. NADH, FAD, and lipofuscin contributions from hMSCs undergoing osteogenic differentiation. Images were taken in the treated (stimulated) and untreated (nonstimulated) groups on days 0, 5, 10, 15, 20. Scale Bar on bottom right corner is 200 μm.

**Figure 7:**
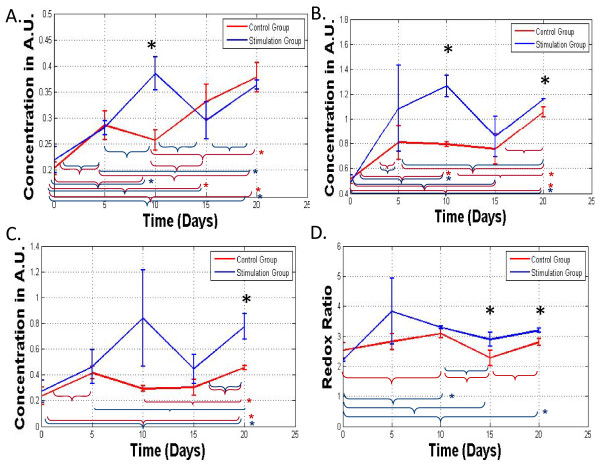
**Metabolic Activity**. (a) NADH, (b) FAD, (c) and lipofuscin fluorescence contributions in arbitrary units (A.U.) vs. time. (d) Metabolic activity was quantified through the redox ratio, FAD/NADH. Statistical significance between groups is marked by one black asterisk (p < 0.05). Statistical differences between time points are marked by a red brace (p < 0.05) and a red asterisk (p < 0.005) for the control group or a blue brace (p < 0.05) and a blue asterisk (p < 0.005) for the stimulation group. The asterisks identify groups that are different even after the stricter Bonferroni criterion is applied, and they, thus, include a subset of the groups identified by braces that are different based on the traditional p value (p < 0.05) used to determine significance.

Over the 20 day differentiation period, increased amounts of NADH and FAD were observed in the TPEF images in both the stimulation and control groups, consistent with the increased cellular activity results from the Alamar Blue studies (Figure 6). Increased contributions from lipofuscin were also observed throughout hMSC osteogenic differentiation, and were most prominent in the stimulation group (Figure 6). Increases in cell number were seen as well in TPEF images between days 0, 5, and 10 in both the control and stimulation groups. Significant enhancements in the levels of NADH and FAD were observed in the stimulation group compared to the control group during the first 10 days of treatment (Figure 7a-c). Indeed, the combination of NADH and FAD contributions to assess the redox ratio reveals changing metabolic activity for both the stimulation and control groups over the 20 days (Figure 7d). On day 15 and day 20, the redox ratio increases statistically significantly in the stimulation group compared to the controls (p < 0.05) (Figure 7d). Interestingly, changes in lipofuscin observed in the stimulation group follow the trend of changes observed in hsp27 expression, suggesting a potential connection with oxidative stress mechanisms.

### Collagen deposition

**Figure 8:**
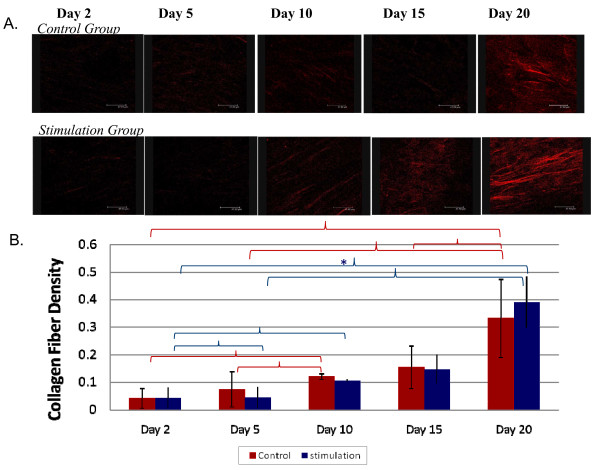
**Protein Deposition**. (a) SHG images examining type 1 collagen deposition in the control and stimulation groups on days 2, 5, 10, 15, and 20. Scale bar is 47.63 um. (b) Quantitative assessment of collagen deposition graphed as collagen density vs. time. Quantitative assessment of collagen deposition is graphed as collagen density vs. time. Collagen density is quantified as the area positive for collagen SHG signal per square micrometer. Collagen fiber is determined as the average fluorescence intensity per pixel from the second harmonic generation images (n = 3). Area (in square micrometers) per pixel is determined by dividing image length and height by total number of pixels in our image. Therefore, collagen density is equal to collagen fiber per pixel divided by the area per pixel. Statistical differences between time points are marked by a red brace (p < 0.05) and a red asterisk (p < 0.005) for the control group or a blue brace (p < 0.05) or a blue askterisk (p < 0.005) for the stimulation group.

Type I collagen protein expression is one of several markers of hMSC-osteoblast differentiation. The lack of centro-symmetry in the collagen fibers results in significant levels of SHG. On day 0 and day 5, fibrillar protein deposition in the control group and stimulation groups was observed (Figure 8a). On day 15, protein deposition by SHG resembled collagen fibers in both the control group and stimulation groups with similar quantitative results (Figure 8b). The rate of change over 20 days in collagen deposition levels is more significant for the stimulation group (p < 0.005), consistent with the observed upregulation of ALP and col I gene expression (Figure 8b).

### Bone mineralization

**Figure 9:**
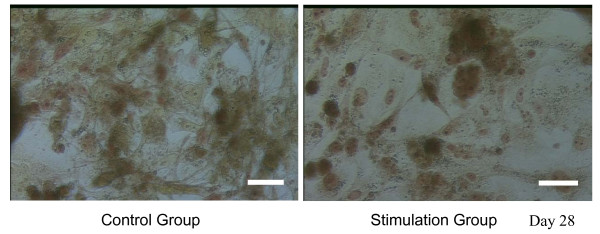
**Calcium Staining**. Alizarin red stain at day 28 showing calcium deposition (red) in both the control group and the stimulation group. Scale bar is 50 um.

On day 28, both the control and stimulation groups exhibited osteogenic differentiation potential towards bone, based on calcium deposition and calcium nodule formation (Figure 9). Calcium staining was compared to a stained negative control of non-differentiated hMSCs (image not shown).

## Discussion

Many studies have revealed increased bone marker expression and increased proliferation on terminally differentiated osteoblasts following exposure to electric fields. For example, in one article, osteoblasts seeded in 3-dimensional constructs and exposed to a 75 ± 2 Hz, 5 ± 1 mV, 2 ± 0.2 mT electric field expressed increased levels of decorin, osteocalcin, osteopontin, type I collagen, type III collagen, TGF-B, and fibronectin [32]. Levels of osteoblast expression markers increased 1.3 times, 12.2 times, 10.0 times and 10.5 times respectively [32]. Another study revealed increased proliferation up to 31% in osteoblasts exposed to a 2-day continuous 1.5 uA/cm2; 3000 Hz electric field [33]. Recent studies have started to reveal responses of hMSCs to osteogenic differentiation in response to electric field stimulation [34-36]. For example, hMSCs isolated from bone marrow and exposed to a 7.5 Hz repetition rate of quasi-rectangular pulses revealed earlier increases in ALP gene expression as well as increases in of Runx2/Cbfa1during the mid stage of differentiation [36]. In addition, calcium content was increased in the pulsed electromagnetic field treated cultures [36]. In another article, human mesenchymal stromal cells exposed to 100 Hz biphasic electric current at 1.5 uA/cm2 induced increased in vascular endothelial growth factor (VEGF) and (BMP)-2 expression [33]. However, in our present study utilizing ostegenic differentiating hMSCs additional assessments and insights are attained. Specifically, in our 28 day study, heat shock protein responses were correlated with osteogenic differentiation markers and field effects on cellular morphology were examined. Cellular activity was tracked as an indication of cell proliferation. Finally, novel optical methods were employed for monitoring specific osteogenic differentiation markers including fibrillar collagen deposition, morphological changes from an elongated hMSC phenotype to a circular osteogenic morphology, and changes in metabolic activity and lipofuscin.

Human mesenchymal stem cells are found in the stromal compartment of bone marrow and are highly proliferative. They are able to differentiate down several lineages including bone, cartilage, fat, and skeletal tissue and play important roles in fracture repair. Specifically, mesenchymal stem cells are a source of progenitors for osteoblast differentiation [37]. Differentiated osteoblasts are important during fracture repair as they contribute to a supply of intramembranous bone formation [37]. Since, previous results have shown that mesenchymal stem cells are the most competent to respond to biophysical input, including electric and electromagnetic fields [14], the application of electric fields on hMSCs may give us better quality of regrown bone and fracture repair.

### Morphological changes

Electric fields are known to alter membrane morphology with regard to cell elongation, alignment, migration, adhesion, and other tissue responses including osteogenic differentiation [38-41]. During the first week of stimulation, namely on day 2 and day 5, eccentricity values describing cellular morphology were 13.2% and 10% lower, respectively, in the stimulation group compared to the control group (Figure 2a, 2b). One possible reason for the immediate morphological change is the extracellular force fields and stress placed on the plasma membrane [42,43].

### Osteogenic response

The differentiation profile of the electrically stimulated group initially revealed a delayed response on days 5 and 10 in ALP and col1 mRNA expression compared to the non-stimulated group, keeping the cells in an undifferentiated state. At day 15, the stimulated group exhibited a 2× higher ALP and col1 expression level than the control group at peak levels. A later but more pronounced effect seen here with osteogenic markers may account for why electrical stimulation is effective and approved by the FDA for healing nonunions [36,44], even though it is not the first line of treatment following average fracture injuries.

### Heat shock response

Heat shock protein expression is upregulated or changed when cells are under stress conditions, including oxidative damage and temperature elevation, for the purpose of maintaining cellular homeostasis [21]. Heat shock proteins participate in protein folding, degradation, and secretion [45]. Furthermore, heat shock proteins may also contribute to differentiation responses. For example, inducing mild heat stress of 41°C for 1 hour can modulate differentiation in normal human epidermal keratinocytes [22]. This effect is not unique to human cell differentiation. Small heat shock proteins, known to have molecular mass between 15 - 30 kDa, such as hsp27 are known to be expressed and regulated during differentiation and development in many organisms including humans, mice, and zebrafish [21]. More specifically, mild heat shock proteins can effect osteodifferentiation [21-23]. In the present study, heat shock protein 27 was upregulated starting on day 10 in the stimulation group (Figure 4a), while osteodifferentiation markers, ALP and col1, were upregulated soon after on day 15 and day 20 (Figure 3a, 3b). Not only did the electric field alter bone marker upregulation, yielding a different time stamp and magnitude on expression levels than in the controls, but the applied field also altered heat shock expression. While hsp27 expression preceded the osteodifferentiation response, recent literature reveals hsp70 involvement with metabolic activity [46-48].

Hsp70, like hsp27, is upregulated during heat and oxidative stress. For example, a sub-lethal heat stress of 42°C on rat hearts in vivo for 15 minutes induced hsp70 and vascular endothelial growth factor (VEGF) upregulation within 4 hours, promoting endothelial cell proliferation [46]. In another study, chondrocytes that had hsp70 mRNA transduced expressed higher metabolic activity under heat stress of 48°C [47]. Mild heat shock altered hsp70 expression in human bone marrow stromal cells and induced cell proliferation, ALP expression, and mineralization [49]. In the present studies, hsp70 expression was downregulated during the first 15 days of stimulation compared to the control group (Figure 4b). The redox ratio reveals metabolic activity was downregulated in the stimulation group on days 15 and days 20 (p < 0.05) (Figure 7d). In the electrical stimulation group, hsp70 increased slightly, with a significant increase by day 20 (Figure 4b). Overall, cellular activity increased in both groups over 20 days based on the Alamar Blue assay (Figure 5). While results show upregulation in hsp27 on days 10, 15, and 20, and upregulation in hsp70 by day 20, the origins of these changes are not yet clear. While differentiation rate and response were affected, the possible relationships between applied electric field, stress response markers, and osteogenic markers, on osteodifferentiation will require further mechanistic insight.

### NADH, FAD, Lipofuscin Contributions

Noninvasive non-linear optical techniques and their combinations, such as second harmonic generation and two-photon excited fluorescence microscopy, provide information that is independent of cell source and environment on several areas that are of interest to tissue engineers, including but not limited to cellular and cell matrix interactions [50]. More specifically, non-linear optical techniques provide information on several aspects of cell and tissue fate including malignancy, cellular differentiation, proliferation, biochemical changes, and apoptosis [51,52]. We utilized a novel quantitative method developed recently [29] to determine the contributions of NADH, FAD and lipofuscin in cells treated with electrical field stimulation. Interestingly, enhancements in the levels of all three chromophores during the first 10 days of treatment, precede the observed upregulation in ALP and Col1 expression and are in agreement with early cellular morphological changes quantified by eccentricity values. The early changes we observe optically via entirely non-invasive means demonstrates the value of such methods as an effective characterization tool over expensive, time consuming biochemical assays and staining.

Over the 20 day differentiation period, we observe a small increase in the redox ratio for both groups, consistent with our previous osteogenic differentiation studies under normoxic conditions [24]. In addition, we find that the redox ratio is significantly higher for the stimulation than the control group. This may indicate that even though the cells are more active, they dedicate a higher percentage of their energy stores towards differentiation-related processes rather than oxidative phosphorylation.

### Cellular mechanisms

**Figure 10:**
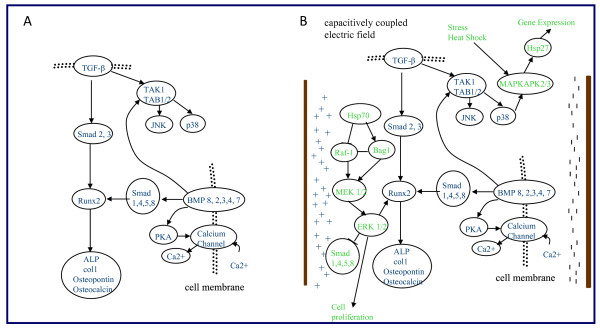
**Cellular Mechanisms during Osteogenic Stem Cell Differentiation**. (a) Osteogenic stem cell differentiation pathways (b) Osteogenic stem cell differentiation pathways and stress response roles in an alternating current electric field.

It is known that BMP, wnt, and TGF-β signaling pathways are involved in upregulating factors such as SMAD, Runx2, ALP, col1, osteocalcium, osteopontin, and kinases JNK and p38 during osteogenic differentiation [53-55]. During osteogenic differentiation, BMP-2,-3,-4,-5 increase SMAD-1, -3, -4, -5, elevating the main transcription factor in osteogenic differentiation, Runx2 (Figure 10a). Runx2/cbfa1 upregulates the early osteogenic marker, ALP, mid marker col1, and late markers osteocalcin and osteopontin (Figure 10a) [53-55]. In addition, it has been demonstrated that osteoblast differentiation is mediated by opposing differentiation and proliferation signaling pathways, specifically ERK1/2 and PKB pathways [56]. The time for complete hMSC to osteoblast differentiation has been demonstrated over 4-5 weeks. In the present studies, 100 ng/mL BMP-2 was included to shorten the time to differentiation by acting directly on the BMP receptor.

Current hypotheses proposing how electric fields affect cell differentiation include altering membrane potential through hyperpolarization and depolarization [57,58], modification of ion channels including density and distribution of receptors, calcium channel activation, and activating the extracellular-signal-regulated kinase (ERK) pathway [38,59]. We suggest two additional factors and their roles in electrical stimulation on osteodifferentiation: heat shock proteins 70 and 27 (Figure 10b).

Hsp70 has been reported to activate ERK1/2 pathways for controlling cell proliferation and survival. ERK1/2 activation can be achieved through Raf-1 and Bag1 activation [60]. Raf-1 and Bag1 upregulate MEK1/2 thus activating the ERK1/2 pathway [53-55,60-62]. The ERK pathway has a direct effect on cell proliferation [53-55,61,62] and was followed through our cellular and metabolic activity assays. Under extracellular stress conditions, heat shock proteins also upregulate mitogen activated protein kinase activated protein kinase 2/3 (MAPKAPK 2/3) upregulating hsp27 [63]. Hsp27 affects gene expression and differentiation, growth, and actin cytoskeleton reorganization [64,65]. In the present studies, hsp27 and hsp70 were upregulated in response to electric field application and compared with differentiation marker response, protein deposition, and cellular activity.

## Conclusions & future directions

Exposing hMSCs undergoing differentiation into osteoblasts in a 20 mV/cm oscillating 60 kHz field for 40 minutes daily over 28 days increased hMSC osteogenic differentiation and activated osteogenic pathways. Future directions for this research include staining and quantifying for ALP and Hsp27 protein expression then correlating ALP and Hsp27 gene expression results to protein expression profiles. Additional transcription factors that are involved in osteogenic differentiation, such as Runx/Cbfa and bone sialoprotein (BSP), will be examined during electric field exposure.

## Competing interests

The authors declare that they have no competing interests.

## Authors' contributions

MHT carried out the experiments, completed data analysis, and drafted the manuscript. WLR developed the algorithms for optical image analysis, assisted in imaging training and was involved in key intellectual discussions. MCG supervised electric field calculations. IG and DLK conceived of and supervised the project. IG and DLK supervised manuscript writing and revisions. All authors read and approved the final manuscript.

## Appendix

### Electric field strength calculations from chamber center

#### Function for Gauss's Law

(for infinite line of charge)

$$\vec{E}\;=\frac{1}{2\pi \varepsilon 0}\left(\frac{\rho }{r}\right)$$

#### Cartesion to Cylindrical Coordinates

X = r cosφ

Y = r sinφ

Z = z

#### Cylindrical to Cartesian Coordinates

$$\text{r }=\sqrt{\left(x^{2}+y^{2}\right)}$$

$$\varphi \;={\text{ tan}}^{-\text{1}}\left(\frac{y}{x}\right)$$

Z = z

#### Convert function from Cylindrical to Cartesian Coordinates

E_x _= Ar cosφ

E_x _= *f*(r) cosφ

$${\text{E}}_{\text{x}\;}=f(\sqrt{\left(x^{2}+y^{2}\right)}\text{) cos}\varphi ;$$

E_y _= A_r _sinφ

E_y _= *f*(r) sinφ

$${\text{E}}_{\text{y}}=f(\sqrt{\left(x^{2}+y^{2}\right)})\text{ sin}\varphi ;$$

E_z _= 0;

Ex=f((x2+y2)) cos (tan−1(yx)) ;

Ey=f((x2+y2)) sin (tan−1(yx));

E_z _= 0;

Trigonometric Identities

$$\text{r }=\sqrt{\left(x^{2}+y^{2}\right)};$$

$$\text{cos }\left({\text{tan}}^{-\text{1}}\text{x}\right)\text{ =}\frac{1}{\sqrt{x^{2}+1}};$$

$$\text{sin }\left({\text{tan}}^{-\text{1}}\text{x}\right)\;=\frac{x}{\sqrt{x^{2}+1}}$$

Replace r with $\sqrt{\left(x^{2}+y^{2}\right)}$

$${\text{E}}_{\text{x}}\;=\frac{1}{2\pi \varepsilon 0}\left(\frac{\rho }{\sqrt{x^{2}+y^{2}}}\right)\left(\frac{1}{\sqrt{{\left(\frac{y}{x}\right)}^{2}+1}}\right)\left(\frac{x}{x}\right)$$

$${\text{E}}_{\text{x}}\;=\frac{\rho }{2\pi \varepsilon 0}\left(\frac{x}{x^{2}+y^{2}}\right)$$

$${\text{E}}_{\text{y}}\;=\frac{1}{2\pi \varepsilon 0}\left(\frac{\rho }{\sqrt{x^{2}+y^{2}}}\right)\left(\frac{y}{x}\right)\left(\frac{1}{\sqrt{{\left(\frac{y}{x}\right)}^{2}+1}}\right)\left(\frac{x}{x}\right)$$

$${\text{E}}_{\text{y}}\;=\frac{\rho }{2\pi \varepsilon 0}\left(\frac{y}{x^{2}+y^{2}}\right)$$

E_z _= 0;

Offset two infinite line charges by distance $[+\frac{d}{2},\text{ -}\frac{d}{2}]$

Ex =ρ2πε0(x−d2(x−d2)2+y2)+ρ2πε0(x+d2(x+d2)2+y2)

Ey =ρ2πε0(y(x−d2)2+y2)+︸ρ2πε0(y(x+d2)2+y2)︸

Find equation for voltage Drop Between Rods

$${\text{V}}_{\text{ab}}=\int Ex•d\ell$$

E field component in the x-direction between two carbon rods

(E field in y-direction is zero)

$${\text{V}}_{\text{ab}}=\int_{\left(\frac{-d}{2}\right)+a}^{\left(\frac{d}{2}\right)-a}\frac{\rho }{2\pi \varepsilon 0}\left[\frac{1}{\left(x-\frac{d}{2}\right)}-\frac{1}{\left(x+\frac{d}{2}\right)}\right]\text{dx}$$

$${\text{V}}_{\text{ab}}=\;\frac{\rho }{2\pi \varepsilon 0}\left(\ln[x-\frac{d}{2}]-\ln[x+\frac{d}{2}]\right)|\begin{array}{l} \left(\frac{d}{2}-a\right)\\ \left(\frac{-d}{2}+a\right) \end{array}$$

$${\text{V}}_{\text{ab}}=\frac{\rho }{2\pi \varepsilon 0}\left(\ln\frac{\left[x-\frac{d}{2}\right]}{\left[x+\frac{d}{2}\right]}\right)|\begin{array}{l} \left(\frac{d}{2}-a\right)\\ \left(\frac{-d}{2}+a\right) \end{array}$$

$${\text{V}}_{\text{ab}}=\frac{\rho }{2\pi \varepsilon 0}\{\left\{\left(\frac{\ln[-a]}{\left[d-a\right]}\right)-\left(\frac{\ln[-d+a]}{\left[a\right]}\right)\right\}$$

$${\text{V}}_{\text{ab}}=\frac{\rho }{2\pi \varepsilon 0}\;\text{ln}\frac{\left[-a][a\right]}{\left[d-a][a-d\right]}$$

Vab= ln−[a^2][d−a][a−d]

Vab=ρ2πε0 lna^2[(d−a)^2]

 Vab=ln(x)^2=2ln(x)

$${\text{V}}_{\text{ab}}=\frac{\rho }{2\pi \varepsilon 0}\;\text{2 }*\text{ ln}\frac{a}{\left[(d-a)\right]}$$

$$\rho =\frac{\text{Vab}\pi \varepsilon \text{0 }}{\text{ln}\left(\frac{a}{d-a}\right)}$$

Substitute for charge density, ρ

$$E_{x}=\frac{V_{ab}}{2\ln\left(\frac{a}{d-a}\right)}\left[\frac{x-\frac{d}{2}}{{\left(x-\frac{d}{2}\right)}^{2}+y^{2}}-\frac{x+\frac{d}{2}}{{\left(x+\frac{d}{2}\right)}^{2}+y^{2}}\right]$$

$$E_{y}=\frac{V_{ab}y}{2\ln\left(\frac{a}{d-a}\right)}\left[\frac{1}{{\left(x-\frac{d}{2}\right)}^{2}+y^{2}}-\frac{1}{{\left(x+\frac{d}{2}\right)}^{2}+y^{2}}\right]$$
